# MicroRNAs in Uteroplacental Vascular Dysfunction

**DOI:** 10.3390/cells8111344

**Published:** 2019-10-29

**Authors:** Xiang-Qun Hu, Lubo Zhang

**Affiliations:** Lawrence D. Longo MD Center for Perinatal Biology, Division of Pharmacology, Department of Basic Sciences, Loma Linda University School of Medicine, Loma Linda, CA 92350, USA

**Keywords:** miRNA, trophoblast invasion, uterine vascular adaptation, preeclampsia, intrauterine growth restriction

## Abstract

Pregnancy complications of preeclampsia and intrauterine growth restriction (IUGR) are major causes of maternal and perinatal/neonatal morbidity and mortality. Although their etiologies remain elusive, it is generally accepted that they are secondary to placental insufficiency conferred by both failure in spiral artery remodeling and uteroplacental vascular malfunction. MicroRNAs (miRNAs) are small no-coding RNA molecules that regulate gene expression at the post-transcriptional level. Increasing evidence suggests that miRNAs participate in virtually all biological processes and are involved in numerous human diseases. Differentially expressed miRNAs in the placenta are typical features of both preeclampsia and IUGR. Dysregulated miRNAs target genes of various signaling pathways in uteroplacental tissues, contributing to the development of both complications. In this review, we provide an overview of how aberrant miRNA expression in preeclampsia and IUGR impacts the expression of genes involved in trophoblast invasion and uteroplacental vascular adaptation.

## 1. Introduction

MicroRNAs (miRNAs) are small non-coding RNAs (~22 nucleotides in length) expressed in plants, invertebrates and vertebrates. These molecules generally regulate gene expression through sequence-specific base pairing with target mRNAs, leading to transcript degradation and translational repression. To date, more than two thousand human miRNAs have been identified [1]. Over 60% human protein coding-genes are targeted by miRNAs [2]. Notably, a single miRNA can target multiple mRNAs and a specific mRNA can also harbor complimentary binding sites in the 3′ untranslated region (3′-UTR) for various miRNAs. Although miRNA research is still in its infancy, the physiological and pathophysiological roles of miRNAs are emerging rapidly. It is clear that miRNAs are involved in virtually every biological process, including cell development, metabolism, proliferation, differentiation, motility and apoptosis. Moreover, the dysregulation of miRNAs is linked with various human diseases such as but not limited to cancer, neurological diseases, and cardiovascular disease [2].

Preeclampsia is characterized by de novo maternal hypertension (>140/90 mm Hg) after 20 weeks of gestation, accompanied by proteinuria or other maternal organ damage or uteroplacental dysfunction [3,4]. Intrauterine growth restriction (IUGR; also known as fetal growth restriction, FGR), characterized as failure of the fetus to achieve his/her genetically determined growth potential, is defined as birth weight less than the 10th percentile [5]. Preeclampsia and IUGR are the most common complications of pregnancy, affecting 3–10% of pregnancies worldwide and being primary causes of maternal and/or fetal/neonatal mortality and morbidity [6,7,8]. Early-onset, but not late-onset, preeclampsia is often associated with increased incidence of IUGR [6,9]. Currently, there is no effective treatment for both complications. Besides their impacts on maternal health and fetal development, these pregnancy disorders also confer an increased risk of cardiovascular diseases later in life [8,10,11]. Moreover, IUGR is often associated with increased incidence of metabolic diseases in adulthood [12]. Etiologies of preeclampsia and IUGR remain unclear. However, the placental dysfunction is known to be essential for the development of preeclampsia and IUGR. The most effective cure for preeclampsia is the delivery/removal of the placenta. The dysfunction of uteroplacental vessels and failure to transform spiral arteries result in reduced placental perfusion, leading to placental insufficiency (uteroplacental vascular insufficiency). Both complications are believed to start from placental insufficiency but with different clinical manifestations [9,13,14].

Despite being a temporary organ developed in the uterus during pregnancy, the placenta expresses many miRNAs including placenta-specific miRNAs. These miRNAs play important roles in placental development and uteroplacental circulation adaptation [15,16,17]. Not surprisingly, dysregulated expression of uteroplacental miRNAs has been demonstrated in both preeclampsia and IUGR, implicating their potential roles in the pathogenesis of these complications [15,18,19]. In this review, we primarily focus on the dysregulation of trophoblast invasion and uteroplacental vascular maladaptation conferred by aberrantly expressed miRNAs in preeclampsia and IUGR.

## 2. MiRNA Expression Patterns in Uteroplacental Tissues

### 2.1. Biogenesis of miRNAs

In human and animal cells, mature miRNAs are produced through a series of enzymatic reactions in the nucleus and cytoplasm. In the nucleus, a miRNA gene is initially transcribed by RNA polymerase II to produce a large miRNA termed primary miRNA (pri-miRNA) in which the mature miRNA sequence is embedded. The pri-miRNA is then processed by a microprocessor complex containing the RNase III enzyme Drosha and its cofactor DiGeorge syndrome critical region 8 (DGCR8). The pri-miRNA is recognized by DGCR8 and cleaved by Drosha. The enzymatic action produces a pre-miRNA with ~70 nucleotides in length. Subsequently, the pre-miRNA is transported to the cytoplasm by exportin-5 with Ran-GTP being a cofactor [20]. Once in the cytoplasm, the pre-miRNA is further processed by Dicer, a ribonuclease III (RNase III), to generate a double-stranded miRNA of ~22 nucleotides.

### 2.2. MiRNA on Gene Expression

The miRNA generated by Dicer is a complementary duplex of miRNA:miRNA*. Whereas miRNA denotes the guide strand (mature miRNA), miRNA* symbolizes the passenger strand. After unwinding by helicases, the miRNA* is degraded, whereas the mature miRNA is loaded onto Argonaute (AGO) protein family (AGO1-4 in human) to form an RNA-induced silencing complex (RISC). Since miRBase release 17, the symbolism miRNA:miRNA* has been replaced with “5p/3p” strand annotation [21]. For a fraction of miRNAs, either of both strands could be loaded to AGOs. The miRNA guides the RISC to its target mRNA by base paring with the complementary sequence located mainly in the 3ʹ-UTR of mRNAs. The ‘seed’ sequence located between nucleotides 2 and 7 of the 5ʹ end of the miRNA is crucial for target site recognition. Consequently, the binding of the RISC to the target mRNA induces transcript degradation and/or translational repression [22,23]. The base-paring between miRNA and mRNA is usually imperfect. Each miRNA may have hundreds of potential mRNA targets, and a given target may also harbor multiple binding sequences for different miRNAs, resulting in a complex regulatory network. Interestingly, some miRNAs are found to activate mRNA translation, although the underlying mechanisms are not fully understood [24,25].

### 2.3. Placental miRNAs in Normal Pregnancy

The human placenta expresses numerous miRNAs to various degree and trophoblasts constitute the major source of placental miRNAs [26,27,28]. Over 600 miRNAs have been identified in normal term human placenta using high-throughput approaches such as miRNA array and next-generation sequencing [29,30]. Similarly, 762 miRNAs are also detected in trophoblasts isolated from the placenta and approximately half of them are notably expressed [27]. Among these miRNAs, chromosome 19 miRNA cluster (C19MC), chromosome 14 miRNA cluster (C14MC) and miR-371-3 cluster are exclusively or preferentially expressed in the human placenta [19]. C19MC harbors 46 genes encoding 58 miRNAs, while C14MC and miR-371-3 cluster consist of 52 and 3 members, respectively. When miRNA expression patterns were examined in placentas or isolated primary trophoblasts from first and third trimester placentas, it appeared that the expression of many placental miRNAs varied with gestational stages. A study using TaqMan array showed that the expression of C19MC increased, whereas the expression of C14MC occurred conversely in trophoblasts as pregnancy advanced [27]. Interestingly, Gu et al. demonstrated that 191 miRNAs were differentially expressed between first and third trimester placentas with 97 downregulated and 94 upregulated in the third trimester using GeneChip miRNA 2.0 array [31]. Further analysis revealed that significant portions of C14MC and C19MC miRNAs as well as the miR-17-92 cluster were downregulated and several members of the let-7 family were upregulated in the third trimester placenta. A subsequent study using miRCURY LNA^TM^ microRNA array showed differential expression of 58 miRNAs between first and third trimester placentas with 41 miRNAs upregulated in term placentas [32]. Among the differentially expressed miRNAs, only 13 of them (miR-10a, miR-29a, miR-29b, miR-29c, miR-101, miR-125b, miR-143, miR-221, miR-377, miR-526b, let-7a, let-7d, and let-7f) were upregulated, whereas miR-675 was the only miRNA downregulated in the third trimester in these two studies. The discrepancies among those studies may result from the lack of standardized protocols, sensitivities of miRNA detection assays, sampling sites of the placenta, gestational ages when samples collected, cell homogeneity/heterogeneity and race/ethnicity, etc. There are also temporal changes for other miRNAs in the placenta. For example, the expression of miR-378a-5p in the placenta was lower in the third trimester than in the first and second trimesters [33]. In contrast, miR-411 and miR-376c levels in the placenta increased progressively over the gestational stages [34,35]. The temporal expression pattern of given miRNAs in the placenta suggest that they may possess stage-specific functions and could play an important role in the placental development.

### 2.4. Placental miRNAs in Compromised Pregnancies

**Figure 1:**
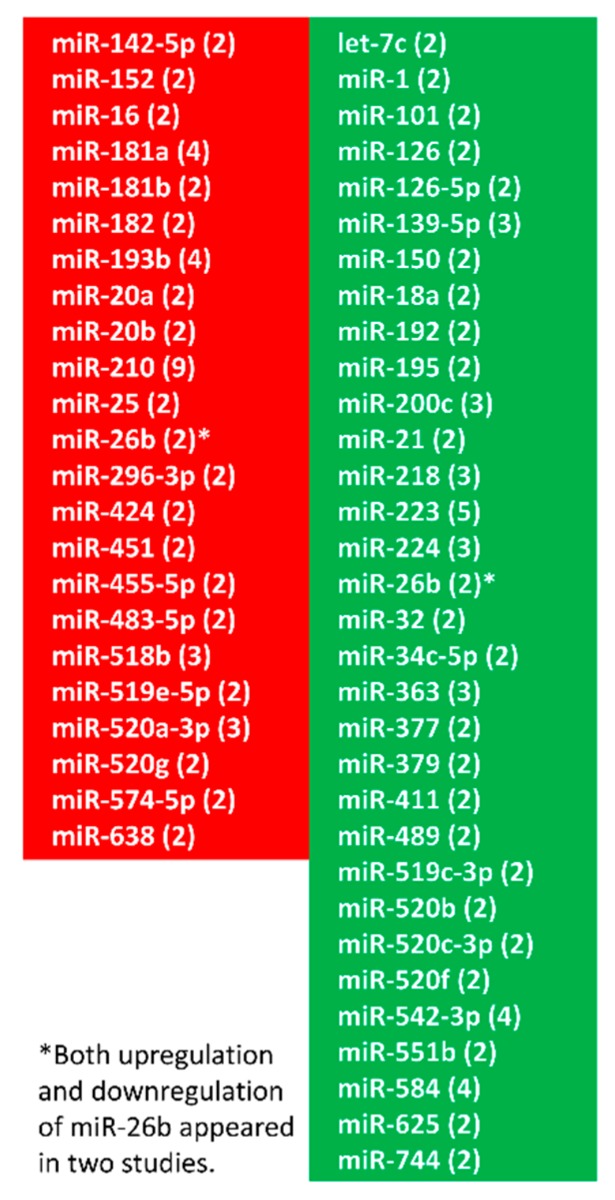
Common members of differentially expressed 304 microRNAs (miRNAs) in preeclamptic placentas detected with high throughout approaches that appeared in at least 2 out of 14 independent studies (see text for detail). MiRNAs in the red box were upregulated and those in the green box were downregulated. Number in parentheses denotes number of studies.

The approaches of miRNA array, high-throughput screening and next generation sequencing have been frequently applied to examine expression profiles of miRNAs in placentas of normal and complicated pregnancies [29,30,36,37,38,39,40,41,42,43,44,45,46,47]. Similar but limited studies using these approaches were also used to profile miRNA expression in IUGR placentas [48,49,50]. These investigations revealed distinct sets of differentially expressed miRNAs (DEMs) between normal and preeclamptic/IUGR placentas. For example, miRNA profiling conducted by Hromadnikova et al. revealed that 11 (miR-515-5p, miR-517-5p, miR-518b, miR-518f-5p, miR-519a, miR-519d, miR-520a-5p, miR-520h, miR-524-5p, miR-525, and miR-526a) and 6 (miR-517-5p, miR-518f-5p, miR-519a, miR-519d, miR-520a-5p, and miR-525) miRNAs of C19MC in preeclamptic and IUGR placentas were downregulated, respectively, compared to placentas of normal pregnancy [51]. Xu et al. [43] observed that 5 of 6 members in miR-17-92 cluster (miR-17, miR-18a, miR-19a, miR-19b, and miR-20a) were downregulated in severe preeclamptic placentas. By examining cardiovascular and cerebrovascular disease associated microRNAs, Hromadnikova et al. [52] demonstrated that placentas from preeclampsia and IUGR requiring termination of gestation before 34 weeks of gestation exhibited downregulation of miR-26a-5p, miR-103a-3p and miR-145-5p and upregulation of miR-499a-5p. They also revealed specific downregulation of placental miR-122-5p, miR-125b-5p, and miR-195-5p in IUGR requiring the delivery before 34 weeks of gestation. Distinct miRNA profiles suggest a role for miRNAs in the pathological processes of preeclampsia and IUGR. Intriguingly, results from these studies varied significantly and sometimes were contradictory. Data pooled from 14 independent studies on miRNA expression in preeclamptic placentas revealed that only a relatively small fraction of DEMs (55 out of 304 miRNAs) changed in the same directions in at least two different studies [29,30,36,37,38,39,40,41,42,43,44,45,46,47] (Figure 1).

The possible causes have been discussed above. Although miRNA profiling approaches are prominent tools to identify DEMs in healthy and pathological conditions, they (especially miRNA array) have their drawbacks such as lower specificity and less quantitative [53,54]. Quantitative reverse transcription polymerase chain reaction (qRT-PCR) is a gold standard technique for accurately quantifying gene expression and is frequently used to validate findings from gene expression profiling. A variety of miRNAs, detected by qRT-PCR, have been found to be aberrantly expressed in preeclamptic and IUGR placentas. A short list of them is presented in Table 1. Interestingly, miR-210 appears to be the most commonly dysregulated miRNA in preeclamptic and IUGR placentas (Figure 1, Table 1). To date, there has been only one study explored miRNA expression in uterine arteries of high-altitude pregnant sheep model which displays mixed features of both early-onset and late-onset preeclampsia. In this study, Hu et al. revealed that miR-210 in uterine arteries of high altitude pregnant sheep was elevated compared to low altitude pregnant animals [55].

## 3. MiRNAs and Uteroplacental Circulation Adaptation under Physiological and Pathophysiological Conditions

The demand of the growing fetus in utero for nutrients and oxygen requires adequate perfusion of the placenta. To accommodate this requirement, maternal cardiovascular system undergoes significant changes as evidenced by ~50% increase in blood volume and cardiac output and a fall in systemic vascular resistance [118]. Dramatic changes also occur locally. For example, uterine blood flow in human and sheep rises from ∼3% of cardiac output in the non-pregnant state to ∼20% at term gestation, corresponding to ~20–50-fold increases in uterine blood flow volume [118]. The increase in uterine blood flow is owing to markedly reduced uterine vascular resistance, conferred by structural and functional changes of uterine arteries in pregnancy.

### 3.1. Structural Adaptation of Uterine Arteries in Normal Pregnancy

Structural adaptation of uterine arteries during pregnancy includes the outward expansive growth of large uterine arteries and remodeling of spiral arteries. One prominent structural change of main uterine, arcuate and radial arteries during pregnancy is the increase in arterial caliber. The diameter of these large arteries increases 2–3-fold in human and various animals [119,120,121,122,123,124,125,126]. These changes are believed to be the results of vascular smooth muscle hypertrophy and hyperplasia stimulated by estrogen [127]. However, the increase in arterial caliber is without changes in the thickness of the vessel wall [119,122]. The increased uterine size due to fetal growth could also passively increase the length of uterine arteries.

The remodeling of spiral arteries occurs in the first half of gestation, fulfilled by extravillous trophoblasts (EVTs) arisen from anchoring villi. The coordinated work by two distinct EVT populations, interstitial EVTs and endovascular EVTs, accomplishes the remodeling process [128,129]. Interstitial EVTs migrate through the uterine stroma, whereas endovascular EVTs migrate in a retrograde manner through the lumen of spiral arteries. The invasion of interstitial EVTs through the uterine interstitium allows them to reach and penetrate the arterial wall, participating in the destruction of arterial media directly or priming vascular smooth muscle cells to be destructed by endovascular EVTs [130,131]. As endovascular EVTs migrate across the inner wall of spiral arteries, they induce apoptosis of endothelial cells and vascular smooth muscle cells [132,133,134], leaving trophoblasts to form the new lining of the lumen and an inert, amorphous fibrinoid material to replace the tunica media. Interestingly, these trophoblasts adopt an endothelial-like phenotype, leading to an apparent re-endothelialization of the vessels [135]. However, it is also suggested that endovascular EVTs promote the migration of vascular smooth muscle cells away from the vessel, but not myocyte apoptosis [136]. Consequently, the vessels become dilated and are converted into flaccid conduits. The mouth of spiral arteries increases from ~200 μm in the non-pregnant state up to 2–3 mm at term of gestation [137]. In addition, the deficit of vascular smooth muscle cells following the remodeling results in loss of responsiveness of spiral arteries to vasoconstrictors.

### 3.2. Functional Adaptation of Uterine Arteries in Normal Pregnancy

Profound functional adaptations also occur in uterine arteries during gestation. Estrogen appears to be a major initiator of these changes mainly through interacting with its receptors [138,139]. In humans, starting from approximately week 9 of gestation, the placental syncytiotrophoblast becomes the predominant source of estrogen during pregnancy [140]. The circulating level of 17β-estradiol (E_2_β) progressively increases over the duration of pregnancy [141]. Coincidentally, uterine blood flow also increases [123,125,142]. A causative role of estrogen and its receptors in pregnancy-induced increase in uterine blood flow was established in a sheep model based on the observations that: 1) a local infusion of an estrogen receptor (ER) antagonist ICI 182,780 into the uterine circulation reduced the increase in uterine blood flow in late ovine pregnancy by 37% [143], 2) prolonged infusion of E_2_β increased uterine blood flow and reduced uterine vascular resistance in non-pregnant, ovariectomized sheep [144], 3) ex vivo treatment of uterine arteries of non-pregnant sheep with E_2_β and progesterone lowered uterine arterial myogenic tone [145], and 4) acute estrogen treatment could also cause vasodilation and increased uterine blood flow [146,147]. Several mechanisms have been shown to contribute to estrogen’s actions in uterine vasculature: 1) acutely stimulating both eNOS activity/NO production in endothelial cells [148,149] and large conductance Ca^2+^ activated K^+^ (BK_Ca_) channel activity in vascular smooth muscle cells [148], and 2) genetically and epigenetically upregulating the expression of endothelial nitric oxide synthase (eNOS), cystathionine γ-lyase (CSE) and BK_Ca_ channel β1 subunit in uterine arteries and subsequently leading to increased NO and H_2_S production as well as enhanced BK_Ca_ channel activity [150,151,152,153,154,155].

The relative contribution of endogenous E_2_β, NO and BK_Ca_ channels on basal uterine blood flow during pregnancy were explored in sheep using pharmacological tools (e.g., ER antagonist ICI 182,780, NOS inhibitor L-nitro-arginine methyl ester (L-NAME), and BK_Ca_ channel blocker tetraethylammonium) [143,156]. It was found that NO negligibly contributed to the regulation of basal uterine blood flow in pregnancy. In contrast, both ICI 182,780 and tetraethylammonium reduced basal uterine blood flow by 40–50%. Similarly, uterine arterial myogenic tone in pregnancy was primarily regulated by BK_Ca_ channels but not by the endothelium [153,157,158]. Thus, pregnancy-induced upregulation of the BK_Ca_ channel is a major determinant conferring the reduction in uterine vascular resistance and increase in uterine blood flow. On the other hand, eNOS probably contributed to attenuated vasocontraction [159,160,161] and enhanced vasodilation in pregnancy [162,163,164,165,166,167] as well as remodeling of uterine arteries (see below). Although pregnancy was found to increase H_2_S biosynthesis in uterine arteries [168], the functional importance of H_2_S in regulating uterine vascular resistance and uterine blood flow remains to be investigated.

Myogenic tone is a major contributor to vascular resistance and plays a critical role in regulating tissue/organ blood flow. Under physiological conditions, the ryanodine receptor (RyR)-BK_Ca_ channel axis is the major mechanism to counter myogenic tone [169]. In vascular smooth muscle cells, BK_Ca_ channels are activated by Ca^2+^ sparks mediated by RyRs to generate spontaneous transient outward (K^+^) currents (STOCs) and the BK_Ca_ channel β1 subunit functions as a primary Ca^2+^ sensor. The Ca^2+^ spark-STOC coupling promotes membrane hyperpolarization and closure of voltage-gated Ca^2+^ (Ca_V_1.2) channels, leading to vasodilation. Uterine arterial myogenic tone decreased in pregnant mice and sheep [170,171,172]. In pregnant sheep, BK_Ca_ channel β1 subunit and ryanodine receptors (RyRs) were upregulated [153,173]. Consequently, Ca^2+^ sparks and STOCs as well as the coupling between these two events were enhanced in uterine arteries in pregnancy [173], thus promoting the attenuation of uterine arterial myogenic tone. These findings are consistent with reduced uterine vascular tone in pregnant sheep [143,174] and reinforced by the observations that the BK_Ca_ channel blocker tetraethylammonium locally infused into the uterine circulation significantly increased uterine vascular resistance and decreased basal uterine blood flow by ~50% in pregnant sheep and had no effect in non-pregnant animals [148,175,176]. Intriguingly, myogenic tone was found higher in uterine arteries of human and rat in pregnancy [177,178] and its implication in pregnancy-induced increase in uterine blood flow is unclear currently. Uterine arteries also displayed both blunted responses to vasoconstrictors angiotensin II and norepinephrine [159,161] and enhanced endothelium-dependent vasorelaxation [162,164,165,166,167]. Ultimately, the structural and functional modifications convert uterine arteries from low-flow, high-resistance to high-flow, low-resistance vessels, allowing to sufficiently perfuse the placenta and to accommodate the demands of the growing fetus. However, failure to structurally and functionally transform uterine arteries would increase uterine vascular resistance and decrease uteroplacental blood flow, leading to pregnancy complications such as preeclampsia and IUGR.

### 3.3. Spiral Artery Maltransformation and Uterine Vascular Maladaptation in Compromised Pregnancies

In normal pregnancy, the remodeling of spiral arteries not only occurs in decidua but also in one-third of myometrium. Brosens et al. first observed that preeclampsia lacks trophoblast invasion of myometrial spiral arteries about half a century ago [179]. This phenomenon is often termed ‘shallow trophoblast invasion’. Impaired trophoblast invasion of spiral arteries in decidua and myometrium were subsequently confirmed in more cases of preeclampsia and IUGR [180,181,182,183,184]. Both preeclampsia and IUGR displayed less media disruption in myometrial vessels [184]. Not surprisingly, the caliber of spiral arteries in decidua in preeclamptic pregnancy was comparable to that in the non-pregnant state (~200 μm) [179]. Similarly, radial arteries in preeclamptic women had a small diameter than those in normal pregnant women [185]. In the IUGR model of eNOS-deficient mice, eNOS deletion impaired pregnancy-induced increase in diameter of uterine arteries [186,187].

The functional adaptation of uterine arteries is also disturbed in preeclampsia and IUGR. Uterine arterial vascular resistance increased, whereas uterine blood flow reduced in both complications [188,189,190,191,192,193]. Increased uterine vascular resistance and reduced uteroplacental blood flow were simulated in a rat model of preeclampsia induced by testosterone injection [194]. Although uterine arterial myogenic tone was not altered in myometrial arteries in preeclamptic patients [195], it was increased in high altitude pregnant sheep and in a rat model of preeclampsia induced by reduced uterine blood flow [196,197]. In addition, flow-mediated relaxation of myometrial arteries was lost in preeclampsia [195]. Moreover, the endothelium-dependent relaxation of myometrial arteries was impaired in preeclampsia [198,199,200,201].

Gestational hypoxia at high altitude is associated with 3-fold increase in the incidence of IUGR and preeclampsia [202,203]. Human pregnancy at high altitude markedly reduced remodeling of decidual spiral arteries [204] and diminished NO-dependent vasorelaxation of myometrial arteries [205]. In Colorado, pregnant women at 3,100 m had 26% smaller uterine artery diameter than their counterparts at 1,600 m [206]. Consequently, pregnancy-induced increase in uterine blood flow was attenuated at high altitude [206,207]. In a sheep model of high-altitude pregnancy, uterine vascular resistance increased owing to increased uterine arterial myogenic tone [172,196]. Aberrant uterine vascular adaptation was also demonstrated in other animal models of IUGR and preeclampsia. In rodent models of preeclampsia induced by surgically induced-reduced uterine perfusion pressure (RUPP) and by testosterone-infusion, uterine arteries displayed increased myogenic tone, enhanced vasoconstriction to phenylephrine and angiotensin II, and reduced endothelium-dependent relaxation [197,208].

### 3.4. Impaired Trophoblast Invasion by miRNAs in Preeclampsia and IUGR

**Figure 2:**
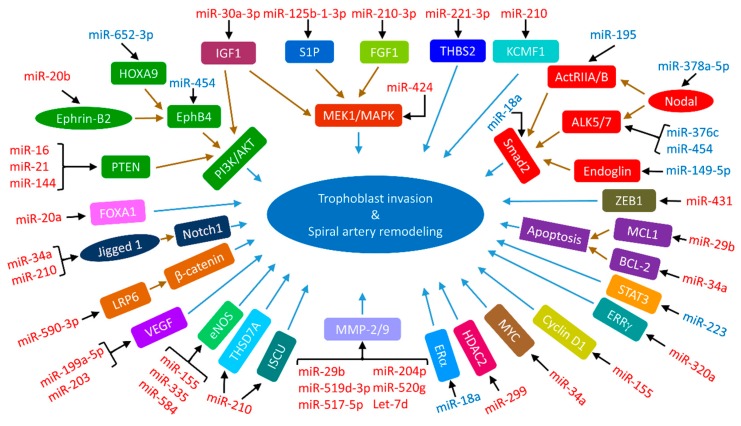
Dysregulated miRNAs and their targets involved in trophoblast invasion in preeclampsia and IUGR. The dysregulation of miRNAs (upregulated miRNA is denoted in red and downregulated miRNA is marked in blue) alters the expression of target gene(s) (linked with a black arrow) of different signaling pathways. Notably, the upregulated miRNA reduces target gene expression, whereas the downregulated miRNA increases target gene expression. In some cases, the miRNA-induced alteration of target gene expression leads to a cascade of signaling events (connected with a brown arrow(s)). Consequently, the altered signaling element(s)/pathway(s) instigates impairment of trophoblast invasion and subsequent failure in modeling of spiral arteries (linked with a blue arrow).

As aforementioned, trophoblast migration/invasion is essential for the transformation of spiral arteries. The migration/invasion of EVTs in the maternal-fetal interface is tightly controlled by various autocrine and paracrine factors such as cytokines, growth factors, and chemokines. These factors activate a variety of signaling pathways including mitogen-activated protein kinases (MAPKs), phosphoinositide 3-kinase (PI3K)/protein kinase B (AKT), JAK-STAT, Wnt, transforming growth factor β (TGF-β), insulin-like growth factor (IGF), NOTCH and other signal pathways [209,210]. Notably, cross-talks may occur between/among different pathways. Activation of these pathways often leads to altered expression of genes controlling trophoblast migration/invasion. Although etiologies of preeclampsia and IUGR are not fully understood, these two pregnancy complications are frequently associated with shallow trophoblast invasion and incomplete spiral artery remodeling [128,182]. MiRNAs are abundantly expressed in the placenta. Comparison among different stages of pregnancy and between normal and compromised pregnancies revealed that some of them are differentially expressed [31,32,50,51,52], suggesting that miRNAs could regulate trophoblast function and invasion via targeting specific genes in both physiological and pathological conditions (Figure 2).

#### 3.4.1. MAPK Signaling Pathway

The MAPK signaling pathway plays an important role in transmitting extracellular signals into cellular responses. Activation of this pathway promotes the transcription of genes that encode proteins involved in the regulation of many cellular processes including differentiation, proliferation, motility and apoptosis [211]. Evidence of the involvement of the MAPK signaling pathway in trophoblast invasion primarily came from the use of pharmacological tools and trophoblast cell lines.

The ERK1/2 MAPK inhibitor U0126 decreased both MAPK phosphorylation and epidermal growth factor (EGF)-triggered HTR8/SVneo cell migration [212,213] or leukemia inhibitory factor (LIF)-induced JEG-3 cell invasion [214]. Blockade of ERK1/2 MAPK and p38 MAPK with U0126 and SB203580, respectively, also suppressed BeWo cell invasion [215] and EGF-stimulated SGHPL-4 cell motility [216]. However, hepatocyte growth factor (HGF)-stimulated SGHPL-4 cell motility was only reduced by ERK1/2 MAPK inhibition but not p38 MAPK inhibition [217]. Preeclampsia was found to be associated with decreased MAPK activation in both placenta and invasive trophoblasts as evidenced by a decrease in the abundance of phospho-MAPK [218,219]. In addition, the catalytic activity of p38 MAPK and level of phospho-p38 MAPK were also reduced in preeclamptic placentas [220]. Huang et al. demonstrated that miR-424 was upregulated in IUGR placentas [116]. MEK1 is the enzyme that catalyzes the phosphorylation of MAPK/ERK. MEK1 encoding gene *MAP2K1* was found to be a target of miR-424 and its expression was negatively correlated with miR-424 levels in IUGR placenta. Both miR-141-5p and phospho-MAPK1 were downregulated in preeclamptic placentas [219]. Activating transcription factor 2 (*ATF2*) was the target of miR-141-5p and inhibiting miR-141-5p increased *ATF2* expression, which would in turn promote the level of dual-specificity phosphatase 1 (DUSP1), an enzyme deactivating MAPK by dephosphorylating the threonine and the tyrosine residues within MAPKs activation site. Thus, downregulated miR-141-5p could indirectly impair phospho-MAPK1 abundance in preeclamptic placentas. Fibroblast growth factors (FGFs) were shown to promote ovine trophoblast invasion via activating MAPK [221]. The expression of *FGF1* in IUGR were repressed by miR-210-3p, which could inhibit trophoblast invasion [115]. Sphingosine-1-phosphate (S1P) is a bioactive sphingolipid metabolite that participates in regulating cell migration, differentiation and survival via interacting its G protein-coupled S1P receptors (S1PRs) [222]. The binding of S1P to the S1PR led to activation MAPK signal pathway and increased production of MMP-2, ultimately promoting invasion of HTR8/SVneo cells [223]. MiR-125b-1-3p level in preeclamptic placentas was elevated [76]. This miRNA was found to target *S1PR1*, resulting in impaired trophoblast invasion. Anton et al. showed that miR-210 inhibited the invasion of primary extravillous trophoblast isolated from first-trimester villous tissue [224]. Intriguingly, the ERK1/2 MAPK inhibitor U0126 ablated miR-210-induced inhibitory effect. This contrasts with the pro-invasive effect of MAPK signaling pathway discussed above. It is not clear whether the use of different cell lines was accountable for the discrepancy.

#### 3.4.2. PI3K/AKT Signaling Pathway

The PI3K/AKT signaling pathway is activated by a variety of extracellular stimuli including EGF and IGF-1. Activated PI3K phosphorylates and activates AKT, which in turn phosphorylates a wide range of substrates involved in cell proliferation, metabolism, survival, and motility [225]. This signaling cascade can be terminated by phosphatase and tensin homolog (PTEN) that dephosphorylates PIP3 and converts it back to PIP_2_ [226]. Like MAPK signaling pathway, the PI3K/AKT signaling pathway is also important for trophoblast invasion [227]. This pathway in trophoblasts appeared to be impaired in preeclampsia [228]. Both preeclamptic and IUGR placentas exhibited reduced expression of miR-16, miR-21, and miR-144 [61,72,113]. *PTEN* appeared to be the direct of miR-16, miR-21, and miR-144 and the downregulation of these miRNAs could enhance PTEN-mediated inhibition on PI3K/AKT signaling pathway and consequently inhibited trophoblast migration and invasion.

#### 3.4.3. TGF-β Signaling Pathway

The TGF-β signaling is involved in regulating many cellular processes including cell proliferation, differentiation, apoptosis, and migration. The TGF-β superfamily of ligands includes TGFβs, activins, Nodal, and bone morphogenetic proteins (BMPs). The TGF-β cascade is initiated by the binding of TGF-β ligands to the type II (ActRIIA, ActRIIB, BMPRII, TβRII and AMHRII) and type I (ALK1–7) receptors that are serine/threonine kinases and phosphorylate and activate receptor-regulated Smads (R-Smads, including Smad1, Smad2, Smad3, Smad5 and Smad8/9). The phosphorylated Smads associate with SMAD4 to form a heterodimeric complex which enters into the nucleus to regulate gene expression [229,230]. In general, activation of this pathway exerts an inhibitory effect on trophoblast migration and invasion [231,232,233,234]. Several components of the TGF-β signaling pathway are targets of aberrantly expressed miRNAs in pregnancy complications. MiR-218-5p expression was suppressed in preeclamptic placentas and miR-218-5p inhibition was found to reduce trophoblast invasion via targeting *TGFB2* [79]. Nodal, ALK5 and ALK7 were all upregulated in preeclamptic and IUGR placentas [233,235]. *NODAL* is a target of miR-378a-5p. Preeclampsia reduced placental miR-378a-5p expression and subsequently relieved *NODAL* repression, resulting in suppressed outgrowth and spreading of extravillous trophoblast cells in first trimester placental explants [33]. Both ActRIIA and ActRIIB were found to be upregulated and miR-195 downregulated in preeclamptic placentas [88,236]. It appeared that dysregulated miR-195-induced upregulation of ActRIIA encoding gene *ACVR2A* and ActRIIB encoding gene *ACVR2B* impaired the invasion of HTR8/SVneo cells. Similarly, both miR-454 and miR-376c, targeting ALK5 encoding gene *TGFBR1* and/or ALK7 encoding gene *ACVR1C*, respectively, were downregulated in preeclamptic placentas and reduced HTR8/SVneo cell invasion and placental explant outgrowth [34,90]. Endoglin, an accessory receptor for TGF-β, negatively regulates trophoblast invasion [237,238]. The expression of endoglin was elevated in preeclamptic placentas and trophoblasts [75,239]. *ENG* is the target of miR-149-5p. The upregulation of endoglin due to downregulation of miR-149-5p in the preeclamptic placenta subsequently inhibited trophoblast invasion [75]. *SMAD2*-targeting miR-18a was downregulated in preeclamptic placenta, thus relieving *SMAD2* repression and impairing trophoblast invasion [43].

#### 3.4.4. Wnt Signaling Pathway

The other important regulator of cell migration is the Wnt signaling pathway. This pathway involves binding of Wnt ligands to the Frizzled (Fz) family of receptors and to low density lipoprotein receptor-related protein 5/6 (LRP5/6) co-receptors, using β-catenin as a transcriptional coactivator to regulate a variety of cellular activity including cell proliferation and migration [240]. Wnt signaling is also crucial for trophoblast invasion [241,242]. Wnt1, β-catenin, and LRP6 were downregulated in preeclamptic placentas [243,244,245]. MiR-590-3p repressed *LRP6* expression by targeting *LRP6* which subsequently reduced β-catenin, MMP-2 and MMP-9 expression, ultimately inhibiting trophoblast migration and invasion [246]. Consistently, downregulation of *LRP6* with siRNA also reduced HTR6/SVneo cell invasion [245].

#### 3.4.5. Notch Signaling Pathway

The Notch signaling pathway plays a pivotal role in regulating cell proliferation, differentiation, migration and apoptosis. The core components of Notch signaling pathway consist of Notch receptors (Notch1-4) and five ligands (Jagged 1/2 and Dll 1, 3, and 4). Ligand binding to Notch leads to cleavage and release of the Notch intracellular domain (NICD), which then travels to the nucleus to regulate transcriptional activity [247]. Notch signaling pathway also participates in placental development [248,249]. The expression of Notch signaling components such as Notch 2/3, Dll 3/4 and Jagged 1/2 were downregulated in preeclamptic placentas [250,251,252,253,254]. Downregulation of Notch 2 with a small hairpin RNA in BeWo cells inhibited both migration and invasion [255]. Both miR-34a and miR-210, which were overexpressed in preeclamptic and/or IUGR placentas [36,95,256], contributed to *NOTCH1* and *JAG1* downregulation that led to impaired trophoblast migration and invasion [97,254,257].

#### 3.4.6. EphrinB2-EphB4 Signaling Pathway

The Eph receptor-interacting protein (ephrin)/erythropoietin-producing hepatocellular (Eph) receptor signaling involves various Eph receptor tyrosine kinases and membrane-tethered ligands [258]. The Eph receptor family consists of nine EphAs and five EphBs. Among them, the ephrin-B2-EphB4 cascade plays a critical role in angiogenesis [259] and is also involved in trophoblast invasion/spiral artery remodeling [260]. Placental EphB4 was increased in preeclampsia, resulting in inhibition of PI3K/AKT signaling pathway [261]. Moreover, enhanced expression of EphB4 was found to suppress trophoblast invasion [262]. MiR-454 was decreased, whereas EphB4 was increased in preeclamptic placenta [89]. *EPHB4* was confirmed to be the target of miR-454 and miR-454 inhibition reduced trophoblast invasion. HOXA9 could directly bind to *EPHB4* promoter and increased the expression of *EPHB4*, and both of them were increased in preeclamptic placentas [263]. Knockdown of *HOXA9* increased migration and invasion of HTR-8/SVneo cells through targeting *EPHB4*, confirming the inhibitory effect of EphB4 on trophoblast invasion. Placental miR-652-3p that targets *HOXA9*, was decreased in preeclampsia [108]. Inhibition of miR-652-3p enhanced *EPHB4* expression and impaired invasion of HTR-8/SVneo cells. However, Zhang et al. showed that *HOXA9* was downregulated in response to elevated miR-210 in preeclamptic placentas [264]. Wang et al. reported that preeclampsia increased miR-20b expression along with reduced level of Ephrin-B2 in the placenta, probably due to Ephrin-B2 encoding gene *EFNB2* being targeted by miR-20b [30].

#### 3.4.7. Insulin-like Growth Factor 1 (IGF-1) Signaling Pathway

The major components of IGF-1 signaling axis include IGF-1/2 and IGF1R. The binding of IGFs to IGFRs initiates the phosphorylation cascade leading to activation MAPK and PI3K/AKT pathways [265]. The IGF-1 signaling pathway is also implicated in placental development [266,267]. Reduced expression of IGF-1 was observed in preeclamptic and IUGR placentas [268,269]. Elevated miR-30a-3p was observed in preeclamptic placenta [68]. *IGF1* was found to be the direct target of miR-30a-3p. Ectopic overexpression of miR-30a-3p suppressed both expression of *IGF1* in HTR-8/SVneo cells and invasion of JEG-3 cells. Upregulated miR-141 in preeclamptic and IUGR placentas targeted a zinc finger protein encoding gene *PLAG1* and subsequently impaired *IGF2* expression, which in turn reduced trophoblast invasion [78,112].

#### 3.4.8. Matrix Metalloproteinases (MMPs)

The invasion of spiral arteries by trophoblasts requires degrading extracellular matrix mediated by MMPs [270]. Among MMPs, MMP-2 and MMP-9 appear to play a crucial role in regulating trophoblast invasion [271]. The expression of MMP-2/9 displayed a temporal pattern: slight dominance of pro-MMP2 over pro-MMP-9 in 6–8 week trophoblasts and supremacy of pro-MMP-9 in trophoblasts from 9 to 11 weeks of gestation [272]. The expression of MMP-2/9 was downregulated in preeclamptic and IUGR placentas [273]. MMP-9 deficiency in pregnant mice simulated clinical features of preeclampsia and IUGR [274]. *MMP2* and *MMP9* are targeted by many miRNAs, which may contribute to the pathogenesis of preeclampsia and IUGR. MiR-20b, miR-29b, miR-517-5p, miR-519d-3p and miR-520g were upregulated in preeclamptic placentas and all of them targeted *MMP2* to suppress trophoblast invasion [67,106,110,111,275]. The invasion of trophoblasts mediated by MMP-9 was impaired by miR-204 [276]. MiR-346 and miR-582-3p regulate endocrine gland-derived vascular endothelial growth factor (EG-VEGF)-induced trophoblast invasion through repressing *MMP2* and *MMP9* [277]. Chemokine CXCL6 inhibited trophoblast cell migration and invasion by suppressing MMP-2 activity in human first-trimester placenta [278]. *CXCL6* was a direct target of miR-519d [279]. The expression of miR-519d was reduced in preeclamptic and IUGR placentas [51], which would then enhance CXCL6′s inhibitory effect on trophoblast invasion. Placental miR-141 was increased in both preeclampsia and IUGR [78,112]. It could suppress trophoblast invasion through targeting the chemokine CXCL12β encoding gene *CXCL12B*, resulting in subsequent *MMP2* downregulation under hypoxia [280]. The increased expression of let-7d in preeclamptic placentas decreased both *MMP2* and *MMP9* expression and suppressed trophoblast migration, although it was not determined whether *MMP2* and *MMP9* were targets of let-7d [58]. Since *MMP-2/9* expression is also regulated by various signaling pathways such as MAPK, PI3K/AKT, Nodal, Wnt and TGF-β signal pathways, miRNAs which disrupted these pathways could also indirectly impaired *MMP-2/9* expression, leading to impaired trophoblast invasion [76,80,223,228,246,281,282].

#### 3.4.9. VEGF

VEGF plays an important role in placenta angiogenesis [283]. In addition to its pro-angiogenesis, VEGF also promotes trophoblast invasion. Using the human EVT cell line SGHPL-4 cells, Lash et al. revealed that VEGF functioned as a chemoattractant to increase trophoblast invasion and motility [284,285]. Several studies showed that miRNAs could target *VEGF* to impair trophoblast invasion. It was reported that expression of miR-199a-5p and miR-203 was increased in preeclamptic placentas [94]. *VEGFA* was the target of miR-199a-5p and miR-203. Both miRNAs inhibited migration and invasion of HTR-8/SVneo cells by downregulating *VEGFA*. Similarly, in a rodent model of preeclampsia, upregulated expression of miR-155 was associated with *VEGF* repression in the placenta [286]. These observations are consistent with the findings of *VEGF* downregulation in trophoblasts of preeclamptic and IUGR placentas [287,288].

#### 3.4.10. eNOS

The involvement of eNOS in regulating trophoblast invasion of uteroplacental artery was initially observed in guinea pig [289]. It was found that the invasion of trophoblasts only occurred when the vessels were dilated by the approaching trophoblasts expression eNOS. Subsequently, eNOS expression was observed in human EVTs [290]. Moreover, it was demonstrated that the endogenously produced NO was responsible for the invasion and motility of trophoblasts using the SGHPL-4 cell line expressing constitutive (eNOS) and inducible isoforms (iNOS) [291]. MiR-155, miR-335, and miR-584 were upregulated in preeclamptic placentas [44,84]. All of them targeted *NOS3* to inhibit both eNOS expression and migration/invasion of HTR-8/SVneo cells. The functional importance of eNOS was further confirmed by the observation that NOS3 knockdown diminished annexin A4 overexpression-induced promotion of cell invasion in both HTR-8/SVneo and JEG-3 cells [228]. Moreover, pregnancy induced less increase in radius of uterine arteries in an eNOS-deficient (eNOS^-/-^) mouse model of IUGR [186,292]. Thus, the dysregulation of *NOS3* by miRNAs like miR-155, miR-335 and miR-584 could also potentially disrupt structural remodeling of large uterine arteries.

#### 3.4.11. Other Signaling Elements

A variety of aberrantly expressed miRNAs in preeclamptic placentas have also found to act on other signaling elements to interrupt trophoblast invasion. STAT3 plays a critical role in trophoblast invasion [293]. The expression of miR-223 and *STAT3* exhibited a reciprocal pattern in preeclamptic placentas: downregulation of miR-223 and upregulation of *STAT3* [294]. *STAT3* was targeted by miR-223 and miR-223 inhibition impaired trophoblast invasion. MiR-18a is downregulated in preeclamptic placentas [295]. An in vitro study revealed that miR-18a targeted ERα receptor encoding gene *ESR1* and miR-18a repression reduced JEG-3 cell invasion [295]. This finding is consistent with the inhibitory effect of estrogen on EVT invasion of spiral arteries during early baboon pregnancy [296]. MYC is a regulator of many genes by binding to enhancer box sequences (E-boxes) [297]. Preeclamptic placentas displayed upregulated miR-34a and miR-34a inhibited trophoblast migration and invasion via targeting *MYC* [69]. The expression of miR-218 was enhanced in preeclamptic placentas and its ectopic expression reduced HTR-8/SVneo cell invasion via targeting *LASP1,* the gene encoding LIM and SH3 domain protein 1 (LASP1) [101]. LASP1 promoted tumor invasion and metastasis by releasing MMPs into the extracellular matrix [298]. It remains to be determined whether this mechanism is involved in miR-218-mediated inhibitory effect on trophoblast invasion. MiR-134 was upregulated in preeclamptic placentas and targeted integrin β1 encoding gene *ITGB1*, resulting in inhibition of trophoblast invasion [299]. Upregulated miR-210 and miR-320a in preeclamptic and IUGR placentas interfered energy metabolism via targeting iron-sulfur cluster assembly enzyme encoding gene *ISCU* and estrogen-related receptor-γ encoding gene *ERRG*, impairing trophoblast invasion [95,300]. Targeting cell cycle regulators by miRNAs could also impair trophoblast invasion. MiR-155 reduced trophoblast invasion via targeting cyclin D1 encoding gene *CCND1* [301]. The upregulated miR-210 reduced the expression of potassium channel modulatory factor 1 (*KCMF1*) and thrombospondin type I domain containing 7A (*THSD7A*) in preeclamptic placentas, resulting reduced trophoblast invasion [96,98]. Upregulated miR-20a in preeclamptic placenta was found to inhibit trophoblast migration and invasion via targeting *FOXA1* [62]. MiR-431, upregulated in preeclamptic placentas, increased E-cadherin via targeting the transcription factor zinc finger E-box-binding homeobox 1 (*ZEB1*) leading to reduced migration and invasion of trophoblasts [105]. Upregulated placental miR-299 in preeclampsia suppressed migration and invasion of trophoblasts via targeting histone deacetylase 2 (*HDAC2*) [102]. Thrombospondin 2 (*THBS2*) was found to repress tumor growth and angiogenesis [302]. Its expression was elevated in preeclamptic placentas due to reduced miR-221-3p expression. Consequently, miR-221-3p inhibitor was found to reduce trophoblast migration and invasion in HTR-8/SVneo cells [81].

Apoptosis of EVTs was increased in early-onset preeclampsia and IUGR, which could in turn impair trophoblast invasion [182,303]. This mechanism appeared to be involved in some miRNAs’ adverse effect on trophoblast migration and invasion in preeclampsia and IUGR. For example, increased expression of miR-29b in preeclamptic placentas could induce apoptosis and reduce of trophoblasts via downregulating myeloid cell leukemia 1 encoded by *MCL1*, an anti-apoptotic member of the B-cell lymphoma 2 (Bcl-2) family of apoptosis-regulating proteins [67]. Similarly, elevated miR-34a in preeclamptic placentas promoted trophoblast apoptosis via repressing anti-apoptotic B-cell lymphoma 2 encoded by *BCL2* [70].

### 3.5. miRNAs in Maladaptation of Uterine Vascular Function in Preeclampsia and IUGR

Besides impaired trophoblast invasion and spiral artery remodeling, the functional adaptation of uterine vasculature is also disrupted in preeclampsia and IUGR. Several important factors involved in the adaptive changes of uterine vascular function are dysregulated in preeclampsia and IUGR. Placental estrogen biosynthesis was impaired due to the downregulation of 17β-hydroxysteroid dehydrogenase 1 (17β-HSD1) and aromatase [29,304,305,306] in these pregnancy complications. Downregulation of BK_Ca_ channel β1 subunit was observed in placental chorionic plate arteries in preeclampsia and uterine arteries in high-altitude pregnancy [172,307]. Concurrently, BK_Ca_ channel-mediated attenuation of uterine arterial myogenic tone was diminished [308]. The expression of CSE was also reduced in preeclamptic and IUGR placentas [114,309]. Placental expression of eNOS remains controversy in preeclampsia and IUGR, downregulation, upregulation or no change were reported [310]. eNOS was downregulated in placental chorionic plate arteries in preeclampsia [311] but upregulated in uterine arteries in high altitude pregnancy [312]. Not surprisingly, circulating E_2_β and hydrogen sulfide (H_2_S) as well as NO bioavailability was reduced in preeclamptic and IUGR pregnancies [304,305,309,313,314,315,316].

**Figure 3:**
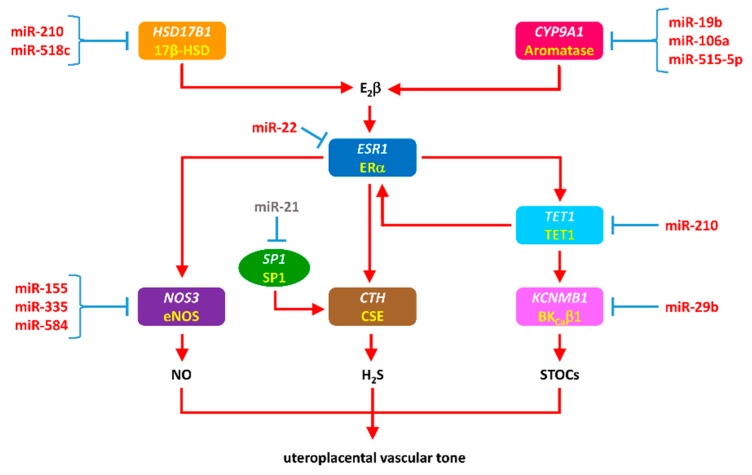
A flowchart showing potential gene targets of aberrantly expressed miRNA on uteroplacental vascular function in preeclampsia and IUGR. During pregnancy, estrogen is predominantly produced in the placenta. 17β-hydroxysteroid dehydrogenase-1 (17β-HSD1) and aromatase are two key enzymes in estrogen biosynthesis. 17β-estradiol (E_2_β) plays a central role in regulating uteroplacental vascular function during pregnancy by enhancing the expression of ten-eleven translocation methylcytosine dioxygenase 1 (TET1), endothelial nitric oxide synthase (eNOS), cystathionine γ-lyase (CSE) and BK_Ca_ channel β1 subunit (BK_Ca_β1). Various aberrantly expressed miRNAs (upregulated miRNA in red) in compromised pregnancies have been show to target genes encoding 17β-HSD1, aromatase, TET1, eNOS, CSE and BK_Ca_β1 (depicted in italics) and decrease the expression of these targets. (Note that both upregulation and downregulation of miR-21 (in grey) have been reported in compromised pregnancies.) The downregulation of these proteins subsequently reduces the generation of E_2_β, nitric oxide (NO), hydrogen sulfide (H_2_S) and spontaneous transient outward currents (STOCs), leading to dysfunction of uteroplacental vasculature.

Accumulating evidence suggest that aberrantly expressed miRNAs contributed to the dysregulation of estrogen biosynthesis, NO signaling, H_2_S signaling and BK_Ca_ channel activity, leading to the maladaptation of uteroplacental circulation in preeclampsia and IUGR (Figure 3). These miRNAs could be either generated locally in the uteroplacental tissues or produced in the placenta and uptaken from the circulation [317]. Circulating microRNAs, either freely or within extracellular vesicles (EVs), could be transported to cells to regulate expression of target genes. For example, placental syncytiotrophoblast EVs (STBEVs) was shown to be internalized into primary human coronary artery endothelial cells and miRNAs inside STBEVs could be transferred into endoplasmic reticulum and mitochondria of the recipient cells, resulting downregulation of specific target genes [318].

Aromatase encoded by *CYP19A1* and 17β-HSD1 encoded by *HSD17B* are two key enzymes in estrogen biosynthesis in the placenta. Aromatase catalyzes the conversion of androstenedione to estrone (E1) and testosterone to E_2_β, whereas 17β-HSD catalyzes the conversion of E1 to the biologically potent E_2_β. Both preeclampsia and IUGR are associated with abnormal steroidogenesis and metabolism. The expression of *CYP19A1* and *17β-HSD1* in the placenta was downregulated in preeclamptic and IUGR pregnancies [29,304,305,319,320], leading to reduced circulating E_2_β [304,305,315,316]. The downregulation of aromatase and 17β-HSD in the placenta was apparently conferred in part by dysregulation of given miRNAs. The expression of miR-19b, miR-106a, miR-210, miR-515-5p and miR-518c was significantly upregulated in preeclampsia and/or IUGR [29,60,93,95]. *CYP19A1* was a confirmed target of miR-19b, miR-106a, and miR-515-5p, whereas *HSD17B* was targeted by miR-210 and miR-518c [29,60,93]. In addition, miR-22 was found to inhibit *CYP19A1* expression indirectly by targeting ERα encoding gene *ESR1* [319]. ERRγ a constitutively active orphan nuclear receptor, stimulated *HSD17B1* expression in the placenta [306]. The expression of *ESRRG* could be inhibited by elevated miR-320a in preeclamptic placenta [103]. Therefore, the dysregulated miR-320a may contribute to *HSD17B1* repression in preeclamptic placenta.

The upregulation of *ESR1* and BK_Ca_ channel β1 subunit encoding gene *KCNMB1* in uterine arteries in pregnancy was mainly achieved through an epigenetic mechanism [321]. Both genes were repressed in uterine arteries of in nonpregnant state due to hypermethylation [154,322]. Elevated E_2_β in normal pregnancy activated ERα in uterine arteries, which subsequently upregulated Ten-eleven translocation methylcytosine dioxygenase 1 (TET1), an enzyme that participates in active DNA demethylation. TET1-initiated demethylation process in turn could promote *ESR1* expression, forming a positively feedback loop [322]. In addition, TET1-induced demethylation also upregulated *KCNMB1* expression in uterine arteries [321]. The expression of *ESR1* in uterine arteries and placentas was reduced in preeclampsia and high-altitude pregnancy [196,322,323]. The expression of *ESR1* could be regulated by miRNAs directly or indirectly. Placental miR-22 was upregulated in preeclampsia and miR-22 binding to the 3′-UTR of *ESR1* could repress *ESR1* expression [319]. Increased miR-210 in uteroplacental tissues was observed in preeclamptic, IUGA and high-altitude pregnancies and *TET1* was a direct target of miR-210 [55,95,174,224]. The downregulation of *TET1* consequently resulted in repression of both *ESR1* and *KCNMB1* [55,174,322]. The downregulation of *ESR1* could in turn lead to the repression of *NOS3* and *CTH*. In addition, *NOS3* and *KCNMB1* could also be directly targeted by given miRNAs. *NOS3* was downregulated in the placenta by upregulated miR-155, miR-335 and miR-584 in preeclamptic pregnancy [44,84]. An abstract presented in Experimental Biology 2018 showed that upregulated miR-29b promoted downregulation of *KCNMB1*, leading to reduced BK_Ca_ channel activity in pulmonary artery smooth muscle cells from patients with idiopathic pulmonary arterial hypertension [324]. *TET1* was also found to be the direct target of miR-29b [325]. Moreover, the expression of CSE encoding gene *CTH* was indirectly inhibited through miR-21-induced downregulation of transcript factor specificity protein-1 (*SP1*) [326]. Given that uteroplacental miR-29b and miR-21 were upregulated in preeclampsia and IUGR [67,113,114,327], it is probably that *NOS3*, *CTH* and *KCNMB1* in uteroplacental vessels are also targeted directly or indirectly by these miRNAs. Owing to their critical roles in regulating uterine vascular function, the downregulation of *ESR1*, *NOS3*, *CTH* and *KCNMB1* would be expected to impair uteroplacental vascular adaptation and to increase uteroplacental vascular resistance, contributing to the development of compromised pregnancies.

## 4. Concluding Remarks

A successful pregnancy is dependent on adequate perfusion of the placenta to supply the fetus with oxygen and nutrients, which relies on appropriate adaptation of uteroplacental circulation. Placental insufficiency caused by inadequate remodeling of spiral arteries and functional maladaptation of uterine vasculature is believed to contribute to the development of both preeclampsia and IUGR. Differentially expressed miRNAs are commonly observed in preeclamptic and IUGR placentas. Increasing evidence suggests that dysregulated miRNAs play important roles in impairing uteroplacental vascular function by targeting numerous genes of multiple signal transduction pathways. Trophoblast invasion starts at ~8 week of gestation and completes in the middle of second trimester. However, current information on the dysregulation of placental miRNAs in preeclampsia and IUGR were mostly obtained from term placentas. MiRNA expression varies with gestational age. Thus, knowledge on placental miRNA expression in the early stage of preeclamptic and IUGR pregnancy is a prerequisite to understand the impacts of dysregulated miRNAs on trophoblast invasion and remodeling of spiral arteries. Moreover, most studies only focused on a specific miRNA and one or a few target genes. As trophoblast invasion and uterine vascular adaptation are regulated by gene regulatory networks, further investigations are required to elucidate key molecular circuits and their coordination/integration that control these processes in normal and compromised pregnancies.

## Figures and Tables

**Table 1:** Aberrant expression of placental miRNAs detected with qRT-PCR in preeclampsia and intrauterine growth restriction (IUGR).

**Preeclampsia**				
**Upregulation**			**Downregulation**	
**miRNA**	**Reference(s)**		**miRNA**	**Reference(s)**
let-7b	[56]		let-7c-5p	[57]
let-7d	[58]		miR-1	[39]
miR-16	[37]		miR-18a	[38,43]
miR-17	[30]		miR-19a-3p	[59]
miR-17-3p	[43]		miR-19b	[43]
miR-19b	[60]		miR-21	[61]
miR-20a	[30,62]		miR-27a-5p	[63]
miR-20b	[30]		miR-34c-5p	[39]
miR-25	[42]		miR-101	[64]
miR-26b	[37,42]		miR-126	[65,66]
miR-29b	[37,67]		miR-127-3p	[57]
miR-31-5p	[63]		miR-133b	[56]
miR-30a-3p	[43,68]		miR-135b-5p	[63]
miR-34a	[69,70]		miR-136-3p	[63]
miR-34a-5p	[71]		miR-139-5p	[39]
miR-92b	[42]		miR-144	[72]
miR-104	[56]		miR-144-3p	[73]
miR-106a	[60]		miR-149	[74]
miR-124-5p	[46]		miR-149-5p	[75]
miR-125b-1-3p	[76]		miR-194	[74]
miR-128a	[56]		miR-195	[43]
miR-137	[77]		miR-218	[43]
miR-141	[78]		miR-218-5p	[79]
miR-142-3p	[77,80]		miR-221-3p	[81]
miR-148a-3p	[63]		miR-223	[43,82]
miR-151	[43]		miR-224	[82]
miR-152	[38]		miR-325	[83]
miR-154-3p	[36]		miR-328	[39]
miR-155	[36,84]		miR-335	[45]
miR-181a	[37,45]		miR-346	[85]
miR-181a-5p	[84,86]		miR-363	[38,45]
miR-182	[36,45]		miR-365a-3p	[63]
miR-182-3p	[36,56]		miR-376c	[34,35]
miR-182-5p	[87]		miR-377	[38]
miR-183	[36]		miR-378a-5p	[33]
miR-193b	[43,63]		miR-379	[43]
miR-193b-3p	[63]		miR-411	[35,38,43]
miR-195	[37,88]		miR-454	[89,90]
miR-197	[42]		miR455-3p	[91]
miR-200b	[36]		miR455-5p	[91]
miR-202-3p	[92]		miR-515-5p	[91,93]
miR-203	[94]		miR-517-5p	[51]
miR-210	[36,38,39,43,63,95,96,97,98,99,100]		miR-518f-5p	[51]
miR-218	[101]		miR-519a	[51]
miR-222	[37]		miR-519a-3p	[57]
miR-296-3p	[42]		miR-519d	[51]
miR-296-5p	[42]		miR-520a-5p	[51,63]
miR-299	[102]		miR-520h	[51]
miR-320a	[103]		miR-524-5p	[51]
miR-335	[37,44]		miR-525	[51]
miR-342-3p	[42]		miR-526a	[51]
miR-362-3p	[104]		miR-532-5p	[57]
miR-423-5p	[57]		miR-539-5p	[57]
miR-424	[45]		miR-542-3p	[38]
miR-431	[46,105]		miR-544	[46]
miR-517-5p	[106]		miR-584	[39]
miR-517a/b	[107]		miR-629-5p	[57]
miR-517c	[107]		miR-652-3p	[108]
miR-518a-5p	[46]		miR-675	[109]
miR-519d-3p	[110]		miR-1301	[82]
miR-520g	[111]			
miR-524	[43]			
miR-584	[44]			
				
**IUGA**				
**Upregulation**			**Downregulation**	
**miRNA**	**Reference(s)**		**miRNA**	**Reference(s)**
miR-10b	[49]		miR-16-5p	[52]
miR-141	[112]		miR-21	[113,114]
miR-193b-3p	[47]		miR-26a-5p	[52]
miR-193b-5p	[47]		miR-100-5p	[52]
miR-210	[95]		miR-103a-3p	[52]
miR-210-3p	[47,115]		miR-122-5p	[52]
miR-221-3p	[47]		miR-125b-5p	[52]
miR-342-3p	[47]		miR-126-3p	[52]
miR-363	[49]		miR-143-3p	[52]
miR-365a/b-3p	[47]		miR-145-5p	[52]
miR-424	[116]		miR-149	[74]
miR-499a-5p	[52]		miR-199a-5p	[52]
miR-519a	[51,117]		miR-346	[85]
miR-574-3p	[47]		miR-515-5p	[48]
			miR-516b	[48]
			miR-517-5p	[51]
			miR-518b	[48,117]
			miR-518f-5p	[51]
			miR-519d	[48,51]
			miR-520a-5p	[51]
			miR-520h	[48]
			miR-525	[51]
			miR-526b	[48]
			miR-1323	[48]
